# Key root traits of Poaceae for adaptation to soil water gradients

**DOI:** 10.1111/nph.17093

**Published:** 2020-12-20

**Authors:** Takaki Yamauchi, Ole Pedersen, Mikio Nakazono, Nobuhiro Tsutsumi

**Affiliations:** ^1^ Japan Science and Technology Agency PRESTO Kawaguchi Saitama 332‐0012 Japan; ^2^ Graduate School of Agricultural and Life Sciences The University of Tokyo Bunkyo Tokyo 113‐8657 Japan; ^3^ Freshwater Biological Laboratory Department of Biology University of Copenhagen Universitetsparken 4, 3^rd^ floor Copenhagen 2100 Denmark; ^4^ UWA School of Agriculture and Environment Faculty of Science The University of Western Australia Perth WA 6009 Australia; ^5^ Graduate School of Bioagricultural Sciences Nagoya University Nagoya Aichi 464‐8601 Japan

**Keywords:** aerenchyma, cortex, drought, flooding, root anatomical traits, stele, wild species, xylem

## Abstract

- Drought and flooding are contrasting abiotic stressors for plants. Evidence is accumulating for root anatomical traits being essential for the adaptation to drought or flooding. However, an integrated approach to comprehensively understand root anatomical traits has not yet been established.
- Here we analysed the root anatomical traits of 18 wild Poaceae species differing in adaptation to a range of soil water content. Regression model analyses revealed the optimal anatomical traits that were required by the plants to adapt to low or high soil water content.
- While the area and number of each root tissue (e.g. stele, cortex, xylem or aerenchyma) were not strongly correlated to the soil water content, the ratio of the root tissue areas (cortex to stele ratio (CSR), xylem to stele ratio (XSR) and aerenchyma to cortex ratio (ACR)) could fully explain the adaptations of the wild Poaceae species to the soil water gradients.
- Our results demonstrate that the optimal anatomical traits for the adaptations to soil water content can be determined by three indices (i.e. CSR, XSR and ACR), and thus we propose that these root anatomical indices can be used to improve the tolerance of crops to drought and flooding stresses.

## Introduction

Global climate change increases the risk of drought and flooding during crop production (Bailey‐Serres *et al*., 2012), and thus the development of novel approaches for crop improvement towards drought and flooding stresses are urgently required. However to achieve this, a better understanding of how wild plant species adapt to the gradients in soil water availability in their natural habitats is needed.

Root anatomical traits strongly influence root trait variations and the balance between resource acquisition and conservation across plant species, and this determines the distribution of plant life (Ma *et al*., 2018; Kong *et al*., 2019). Roots have three radial cell layers (epidermis, cortex and endodermis; outer to inner layers) that encircle the stele (Coudert *et al*., 2010; Petricka *et al*., 2012), and most plant species have several cortical cell layers in their roots (Armstrong, 1979; Justin & Armstrong, 1987). Water and nutrients that are taken up by the roots are transported to the leaves through the xylem vessels in the stele (Kong *et al*., 2021). By contrast, cortical cells consume water and nutrients by respiration, and thus root structural allometry determines the balance between carbon supply and consumption (Kong *et al*., 2021). However, the formation of aerenchyma (i.e. large internal gas spaces) in the cortex reduces the metabolic costs for roots during flooding (Armstrong, 1979). Moreover, the aerenchyma facilitates internal oxygen diffusion from the shoot to the root tips in flooded, anoxic soils (Colmer & Voesenek, 2009; Yamauchi *et al*., 2018; Pedersen *et al*., 2021). Many previous studies have demonstrated that the development of the xylem and aerenchyma in the roots are both essential for crop tolerance to drought and/or flooding (Colmer, 2003; Lynch, 2018). The adaptive trade‐off between water transport through the xylem vessels and oxygen supply through the aerenchyma has not been widely considered (Yamauchi *et al*., 2021).

Recently, we have shown that higher cortex to stele ratio (CSR) and aerenchyma to cortex ratio (ACR) in the roots of wetland species of rice (*Oryza sativa*) than those of upland species of maize (*Zea mays* ssp. *mays*) and wheat (*Triticum aestivum*) are associated with their requirements for soil water (Yamauchi *et al*., 2019). Thicker rice roots having larger cortical areas can also transport more oxygen into the root tips than thinner rice roots having smaller cortical areas (Yamauchi *et al*., 2019). High proportions of cortex and aerenchyma coordinately support the adaptation of wetland species to anoxic conditions in flooded soils. Another line of evidence has demonstrated that anoxia or hypoxia in the stele restricts the loading of essential ions into the xylem (Gibbs *et al*., 1998; Colmer & Greenway, 2011; Kotula *et al*., 2015). Radial oxygen profiles indicate that there are high oxygen levels in the porous cortex and much lower levels of that of the dense stelar tissues (Armstrong *et al*., 1994; Gibbs *et al*., 1998), and thus the available oxygen within the stele is insufficient to support aerobic respiration under hypoxic conditions (Gibbs *et al*., 1998). A larger stelar size may increase the risk of developing larger ‘anoxic’ cores in the roots when in flooded soils (Armstrong *et al*., 2019). These evidences suggest that multiple anatomical traits are simultaneously required for flooding tolerance.

For drought tolerance, the formation of aerenchyma also helps reduce the respiratory costs of roots in deeper rooting maize lines, leading to the stimulation of root growth under water stress in dry soils (Zhu *et al*., 2010). Moreover, larger xylem and stelar areas in the roots of an upland rice were proposed to be adaptive traits for drought avoidance in field conditions (Uga *et al*., 2008). By contrast, a wheat variety having smaller xylems is more tolerant to drought than that of a variety with larger xylems (Richards & Passioura, 1989). Although there is accumulating evidence for the important role of root anatomical traits with respect to drought tolerance, the coordination of multiple root anatomical traits remains unclear.

In this study, we aimed to identify the key root anatomical traits for the tolerance to drought and flooding in a diverse range of wild Poaceae species. We measured the root tissue areas and analysed the ratio of the adventitious root tissues of 18 species of wild Poaceae that differed in preferences to soil water. Moreover, we conducted principal component and modelling analyses to reveal the relationships of the root anatomical traits with the soil water content.

## Materials and Methods

### Field survey of wild Poaceae species

**Fig. 1:**
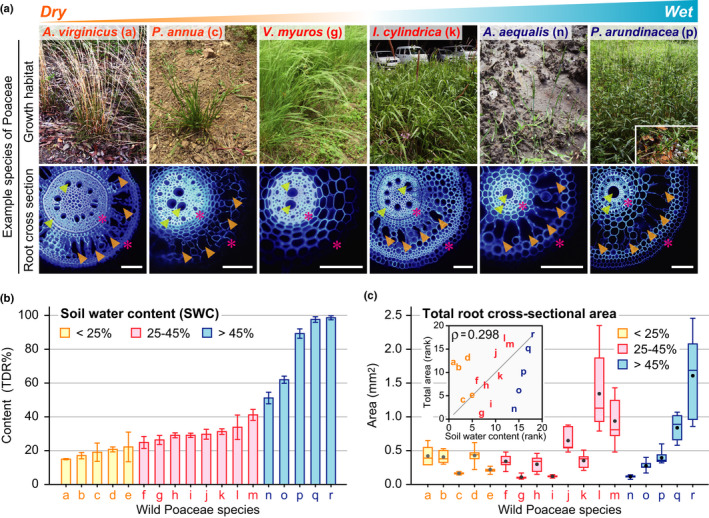
Root anatomical traits of the wild Poaceae species as related to their natural habitats and the soil water gradients. (a) Example photographs of six of the 18 target species and cross‐sections taken from their adventitious roots at 10 mm from the root–shoot junction of adventitious roots. The endodermis and epidermis, as detected under UV light, are indicated by magenta asterisks. Lysigenous aerenchyma and xylem vessels are indicated by orange and yellow arrowheads, respectively. Bar, 100 μm. (b) Soil water content (SWC) at a depth of 10 cm below the soil surface in the surroundings of each species. Values are means ± SD (*n* = 3). (c) Total cross‐sectional areas at 10 mm from the root–shoot junctions of the adventitious roots of the wild Poaceae species. The inset graph shows the result of Spearman's rank correlation test (*ρ*‐value) between the SWC and total root cross‐sectional area. Boxplots show the median (horizontal lines), 25^th^ to 75^th^ percentiles (extension of the boxes), minimum to maximum values (error bars) and mean values (dots in the boxes) (*n* = 6–9). The species represented by letters a–r are as defined in Table 1.

The field survey was carried out in Nagoya city, Aichi prefecture, Japan, from May 2015 to July 2015. A total of 18 target species of wild Poaceae were identified by their morphological features, such as their panicles and stem trichomes (Fig. 1a, Supporting Information Fig. S1a; Table 1). The classification of the subfamilies was in accordance with Soreng *et al*. (2015). The soil water content (SWC) at a depth of 10 cm below the soil surface surrounding the target plants was measured by time domain reflectometry (TDR) soil moisture meter (TDR150; Spectrum Technologies Inc., Aurora, IL, USA) following the manufacturer's instructions (Fig. S1b). The measurements were conducted three times at three randomly chosen positions in a 10 cm radius around the plants, and mean values were used as the SWC of the target plants (Fig. S1b). A total of 13 species were found in sandy‐clay soil, whereas five were found in clay soil in paddy fields (species ‘n’ to ‘r’ in Table 1). The panicles or leaves of the sampled plants were photographed with a scale to support the discussion of the effect of plant height. Subsequently, target plants were excavated from the soils and their roots were thoroughly washed. Whitish young adventitious roots (100 mm to 150 mm lengths; six to nine replicates) from each one individual were sampled (Fig. S1c). Sampling of adventitious roots of the 18 wild Poaceae species were conducted after three continuous days without rainfall (Table 1). For four species, we subsequently sampled the adventitious roots from other individuals at the same locations after three rainy days to compare root anatomical traits under conditions of different SWC (Table S1; Zhou *et al*., 2001).

**Table 1:** Soil water content in the surrounding of the 18 wild Poaceae species and the lengths of the adventitious roots subjected to the root anatomical observations.

Letter	Subfamily	Species	Soil water content (%)^a^	Adventitious root length (mm)^b^
a	Panicoideae	*Andropogon virginicus*	15.5 ± 0.4	71.9 ± 16.7
b	Pooideae	*Lolium perenne*	17.6 ± 1.8	71.9 ± 14.8
c	Pooideae	*Poa annua*	19.6 ± 5.4	67.0 ± 10.6
d	Pooideae	*Agropyron ciliare*	21.3 ± 1.5	66.7 ± 11.1
e	Pooideae	*Lolium multiflorum*	22.8 ± 8.8	78.8 ± 11.7
f	Pooideae	*Bromus catharticus*	25.4 ± 3.6	72.1 ± 8.4
g	Pooideae	*Vulpia myuros*	26.9 ± 2.7	68.3 ± 10.4
h	Panicoideae	*Setaria viridis*	29.6 ± 2.7	94.2 ± 21.0
i	Pooideae	*Briza minor*	29.7 ± 1.3	68.4 ± 13.0
j	Panicoideae	*Setaria faberi*	30.3 ± 2.9	59.1 ± 8.5
k	Panicoideae	*Imperata cylindrica*	31.9 ± 1.6	67.0 ± 15.8
l	Panicoideae	*Sorghum halepense*	34.4 ± 7.3	93.1 ± 22.5
m	Panicoideae	*Echinochloa crus‐galli*	41.7 ± 3.2	70.9 ± 16.2
n	Pooideae	*Alopecurus aequalis*	51.7 ± 3.4	71.9 ± 13.4
o	Pooideae	*Beckmannia syzigachne*	62.6 ± 2.1	78.5 ± 11.7
p	Pooideae	*Phalaris arundinacea*	89.9 ± 2.8	96.8 ± 26.0
q	Panicoideae	*Echinochloa oryzoides*	98.3 ± 1.7	68.8 ± 13.0
r	Arundinoideae	*Phragmites australis*	99.3 ± 1.2	74.3 ± 33.8

Lower‐ (< 25–), middle‐ (25–45%) and higher‐SWC (> 45%) groups are indicated by the different colour fonts.

^a^ Values are means ± SE (*n* = 3).

^b^ Values are means ± SE (*n* = 6–9).

### Measurement of the root anatomical traits

Root cross‐sections were prepared at 10 mm below the root–shoot junction of adventitious roots. These sections were prepared by hand using a razor blade and immediately placed on glass slides and each section was photographed using an optical microscope (BX60; Olympus, Tokyo, Japan), with a CCD camera (DP70; Olympus). The boundary of the stele and cortex, i.e. endodermis, was detected using ultraviolet (UV) irradiation (Fig. S1d). Aerenchyma lacunae were defined as collapsed cortical cells (Fig. S1d). The living cortex area was calculated by subtracting the area of aerenchyma from the total cortical areas. Outlines of the tissues in the cross‐sectional images were traced by freehand selection and their areas were quantified using Imagej software (v.1.43u; National Institutes of Health, Bethesda, MD, USA).

### Principal component analysis

Principal component analysis (PCA) was performed on the number and area (both standardized) of the root tissues using the prcomp function in the ‘stats’ package in R software (v.3.5.2). Standard deviations, proportion of variances and loading scores of the first two principal components are shown in Table S2.

### Regression model analysis

Fitting of the linear and nonlinear models for each root anatomical index and SWC was conducted using the standard statistical tools in R software using the lm function for linear and nls function for nonlinear models. The best models were determined by referencing the Akaike Information Criterion (AIC) and Bayesian Information Criterion (BIC) values, as calculated using the standard statistical tools in R software. Subsequently, nonparametric confidence intervals of the parameters were obtained using the bootstrap (nlsBoot) function in the nlstools package in R software, and 1000 bootstrap replicates were run. The models were selected based on the mean values of the bootstrap replicates.

### Statistical analysis

Spearman’s rank correlation coefficient (*ρ*‐value) and its significance level (*P*‐value) were calculated for the root tissue areas or the ratio of tissue areas to SWC, using the standard statistical tool for rank correlation (cor.test) function in R software.

## Results

### Field survey of the soil water content in the natural habitats of the wild Poaceae species

A total of 18 species of wild Poaceae were identified in their natural habitats based on their morphological features (Fig. 1a). Most of the species belonged to the subfamilies Pooideae and Panicoideae, while a wetland species, *Phragmites australis*, belonged to the subfamily Arundinoideae (Table 1). The range of the mean SWC varied from 15.5% to 99.3% *v/v*, and the species were ordered from ‘a’ to ‘r’, according to their SWC levels (Fig. 1b; Table 1). The lower‐SWC group (SWC < 25%) included the dryland species *Andropogon virginicus* ‘a’ and *Lolium perenne* ‘b’, and the higher‐SWC group (SWC > 45%) included wetland species *Phalaris arundinacea* ‘p’ and *Phragmites* *australis* ‘r’ (Fig. 1b; Table 1). These results indicate that our classifications were reasonable with respect to the soil water requirements.

### Analysis of root tissue areas

The total root cross‐sectional area (Fig. 1c) and the area of each root tissue (i.e. stele, cortex, xylem, aerenchyma and living cortex; Fig. S2), and the numbers and average areas of the xylem and aerenchyma (Fig. S3) were measured using the root cross‐sections of the 18 wild Poaceae species. We found no significant correlations for the SWC and areas or numbers of root tissues except for a weak correlation between the total area and average area of the aerenchyma (Figs S2, S3). Among the 18 species, *Sorghum halepense* ‘l’ and *Phragmites* *australis* ‘r’ had the higher plant heights (Fig. S4a) and larger total root cross‐sectional areas (Fig. 1c), whereas *Poa annua* ‘c’, *Briza minor* ‘i’, *Alopecurus aequalis* ‘n’ and *Beckmannia syzigachne* ‘o’ had the lower plant heights (Fig. S4b) and smaller total root cross‐sectional areas regardless of their preferences for SWC (Fig. 1c). Moreover, almost all of the species having middle plant heights (Fig. S4a) had the middle total root cross‐sectional areas (Fig. 1c). These results suggest that variations of the plant heights resulted in noise in the correlations between the root anatomical traits and the SWC.

### Principal component analysis of the root anatomical traits

To investigate the interaction among the root anatomical traits, PCA was performed on all of the root anatomical traits (Fig. S5a). The result showed that the primary axis was explained by all of the root anatomical traits (principal component one (PC1), 69%) and the secondary axis was explained by the trade‐off between the cortical (aerenchyma) and stellar (xylem) areas and numbers (principal component two (PC2), 16%; Fig. S5a; Table S2). The result also suggested that the primary axis was associated with the plant height (Figs S4, S5a) and the secondary axis was weakly associated with adaptation to the soil water gradients (Figs 1, S5a). Subsequently, PCA was performed on the areas of the stele, cortex, xylem and aerenchyma (Fig. S5b). We found that the areas of these four tissues were enough to explain the interactions of the root anatomical traits with respect to the plant height (PC1, 77%) and adaptation to the soil water gradients (PC2, 19%; Figs 1, S4, S5b; Table S2). However, the bipolar effects of aerenchyma formation on the adaptations to the dry and wet soil were not captured by the PCA, and this might lead to underestimation of PC2 for the lowest‐SWC species (‘a’ to ‘c’ in Fig. S5b).

### Analysis of the ratio of root tissue areas

**Fig. 2:**
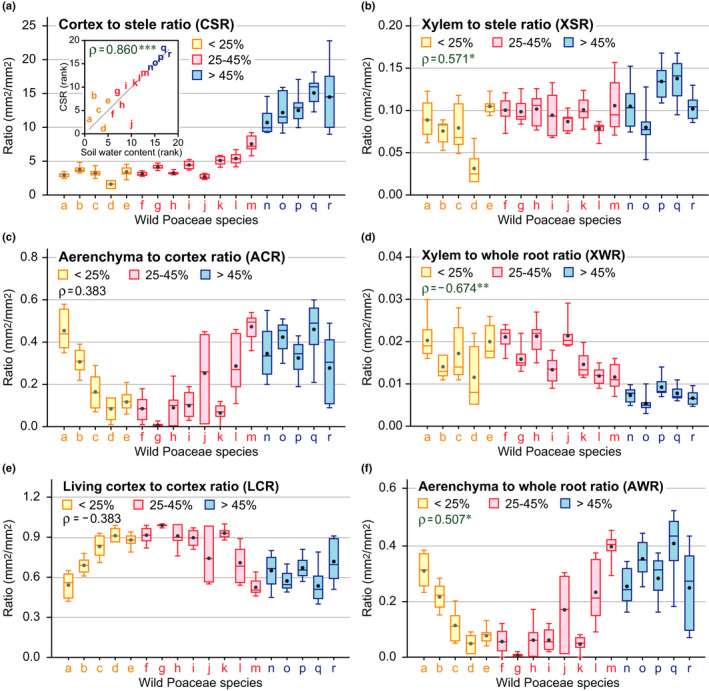
Ratio of the tissue areas in the roots of the 18 wild Poaceae species. Cortex to stele ratio (CSR) (a), xylem to stele ratio (XSR) (b), aerenchyma to cortex ratio (ACR) (c), xylem to whole root ratio (XWR) (d), living cortex to cortex ratio (LCR) (e) and aerenchyma to whole root ratio (AWR) (f) at 10 mm from the root–shoot junctions of the adventitious roots of the wild Poaceae species. The inset graph shows the results of Spearman's rank correlation test (*ρ*‐value; ***, *P* < 0.001) between the soil water content and CSR (a). The results of the Spearman's rank correlation tests (*ρ*‐value; **, *P* < 0.01; *, *P* < 0.05) between the soil water content and the ratio of each root tissue area are shown in the upper left of each graph (b–f). Boxplots show the median (horizontal lines), 25^th^ to 75^th^ percentiles (extension of the boxes), minimum to maximum values (error bars) and mean values (dots in the boxes) (*n* = 6–9). The species represented by letters a–r are as defined in Table 1.

To compensate for the bias caused by the variations in the plant heights and to detect the bipolar effects of the aerenchyma formation, the ratio of the four root tissue areas (CSR, xylem to stele ratio (XSR) and ACR) were calculated and then correlated to the SWC (Fig. 2a–c). CSR showed a strong positive correlation with SWC (Fig. 2a); and XSR was also positively correlated with SWC (Fig. 2b). Although we did not observe significant correlations between ACR and SWC, the ACR was higher in the species adapted to the lowest or highest SWC (Fig. 2c). In contrast to the XSR, the xylem to whole root (i.e. total root cross‐sectional area) ratio (XWR) was negatively correlated to the SWC (Fig. 2d). The living cortex to cortex ratio (LCR) was inversely correlated to the ACR, and it was lower in the species adapted to lowest and highest SWC (Fig. 2e). A higher CSR in the species adapted to higher SWC lead to an increase in the proportion of the cortex in which the aerenchyma was formed. The aerenchyma to whole root ratio (AWR) was positively correlated with the SWC, even though the AWR was also high in the species adapted to a low SWC (Fig. 2f).

### Regression model analyses of the ratio of the root tissue areas

**Fig. 3:**
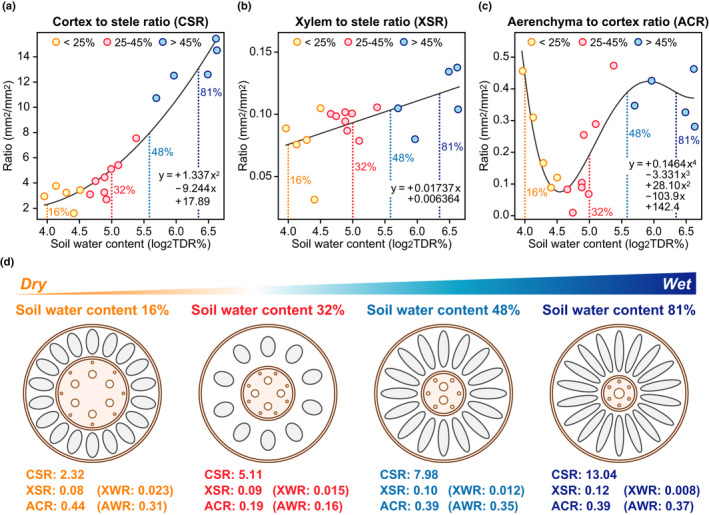
Adaptive root anatomical traits of the wild Poaceae species to the gradients in the soil water. (a) Nonlinear regression analysis of the soil water content (SWC; log_2_TDR%) and cortex to stele ratio (CSR) of the 18 wild Poaceae species. The solid line shows the model obtained by the quadratic curve fitting. (b) Linear regression analysis of the SWC and xylem to stele ratio (XSR). The solid line shows the model obtained by linear fitting. (c) Nonlinear regression analysis of the SWC and aerenchyma to cortex ratio (ACR). The solid line shows the model obtained by quartic curve fitting. The models were tested by bootstrap resampling (*n* = 1000) and the mean values for each term of the equations are shown (a–c). The coloured dots indicate the mean values for the CSR, XSR and ACR. (d) Adaptive anatomical root traits for the soil water gradients, as predicted by the models of CSR (a), XSR (b) and ACR (c). The xylem to whole root ratio (XWR) and aerenchyma to whole root ratio (AWR) were calculated by using the values of the CSR and XSR, and CSR and ACR, respectively (d). The width of the endodermis and epidermis were not considered for the calculation.

To predict the optimal anatomical structures of the roots for the adaptation of the wild Poaceae species to the soil water gradients, regression models were constructed using the mean SWC and all datasets for the CSR, XSR and ACR (Fig. S6). Based on the values of the AIC and BIC, the quadratic and linear models were respectively selected for the CSR (Fig. S6a,b) and XSR (Fig. S6c,d). For the ACR, we selected the quartic model as the fitted pattern was reasonable for the adaptation of the wild Poaceae species, and the AIC and BIC values were lower for the quartic model than those for the linear, quadratic and cubic models (Fig. S6e–h). Due to the polynomial fit, we also acknowledge that our model is only valid within the observed range of SWC (15.5% to 99.3%). Subsequently, nonparametric confidence intervals of the selected models were calculated from the bootstrap replicates, and the mean values of the replicates were selected as the best models for CSR, XSR and ACR (Fig. 3a–c). Based on the equations obtained by the regression analyses, adaptive CSR, XSR and ACR values in the representative SWC (i.e. 16%, 32%, 48% or 81%) were calculated (Fig. 3d).

### Responses of the root anatomical traits to intermittent rainfall

To evaluate whether our models represent the adaptations of wild Poaceae species to soil water gradients or if it also represents phenotypic plasticity within the species in response to the soil water gradients, we investigated the response levels of the root anatomical indices (i.e. CSR, XSR and ACR) to the increased SWC caused by three‐days of intermittent rainfall (Fig. S7; Table S1). We compared the actual values in the roots of four species randomly sampled after three nonrainy days with those sampled after three rainy days with the calculated values obtained from the regression models (Fig. 3a–c). The slopes of all the lines between the actual values before and after the rainfall were less than those between the ideal values (Fig. S7). This indicates that the phenotypic plasticity (response/acclimation) of the root anatomical traits was smaller than the species‐level differences (adaptation). We therefore conclude that the models developed represent the key root anatomical traits required to adapt to the soil water gradients.

## Discussion

In this study, the root anatomical traits of 18 wild Poaceae species in their natural habitats with gradients of soil water were investigated (Figs 1, S1; Table 1). We found no strong correlations between the root tissue area and number of roots and the SWC (Figs S2, S3). Subsequent PCA revealed that the root tissue area correlated to the plant height of the wild Poaceae species (Figs S4, S5). This result is supported by the previous finding that plant height of grasses correlates positively with the cross‐sectional areas of root, stele and xylem (Wahl & Ryser, 2000). PCA also revealed a trade‐off between the cortex and stele as an adaptation to the dry and wet soils (Fig. S5b). CSR showed a strong positive correlation with SWC (Fig. 2a). This suggests that having a larger CSR is an advantage for plants growing under low SWC. Xylem vessels transport water from the soil, whereas cortical cells consume water by respiration (Kong *et al*., 2021). This suggests that having a smaller cortex reduces the metabolic cost of root elongation in order to acquire soil water at deeper levels under dry soils. By contrast, oxygen deficiency in the stelar cells during flooding was found to restrict the loading of the nutrients into the xylem (Colmer & Greenway, 2011). The proportion of the stelar area within the total cross‐sectional area was smaller in the wetland species than that in the dryland species (McDonald *et al*., 2002). This supports the idea that higher CSR, which is associated with a smaller stele and larger cortex ratio, is more adaptive to high SWC.

Unexpectedly, XSR positively correlated to SWC (Fig. 2b), whereas the dryland species having a smaller CSR showed a higher XWR, when compared with the wetland species (Fig. 2d). While a higher CSR associated with a smaller stele would be a disadvantage for the growth of a wetland species, a higher XSR may partly compensate for the lower CSR in the roots (Fig. 2a,b). In addition to the higher XSR, an increase in the number of roots could be a key factor to compensate for the less effective transport of water and nutrients in each root of the wetland species (Visser *et al*., 1996; Yamauchi *et al*., 2019).

Although we did not observe any significant correlation between ACR and SWC, the ACR was higher in species adapted to the lowest or highest SWC (Fig. 2c). This clearly shows that the aerenchyma formation is an essential trait for adaptation to both drought and flooding. The aerenchyma formation contributes not only to the internal oxygen diffusion under flooded conditions, resulting in a supply of oxygen to the roots in soil anoxia, but also to reduce the respiratory costs and stimulate root growth in drought and flooding (Armstrong, 1979; Lynch, 2015). In line with these findings, LCR was much lower in species adapted to both the lowest and highest SWC (Fig. 2e). A higher CSR has been reported in the roots of the rice than that of the maize and wheat leading to an increase in the proportion of the cortex (Yamauchi *et al*., 2019). Thus, due to the higher proportion of the cortex, the AWR in the rice roots also showed an increase (Yamauchi *et al*., 2019). Indeed, we observed a positive correlation of AWR and SWC (Fig. 2f) but not with ACR and SWC (Fig. 2c). These results support the idea that a higher CSR helps expand the aerenchyma in the roots of the wetland species.

To predict the optimal root anatomical structures that are conserved among the wild Poaceae species for adaptation to soil water gradients, a regression model was constructed using SWC and CSR, XSR or ACR (Fig. 3a–c). The adaptive root model for low SWC (16%) showed that a low CSR (2.32) with low XSR (0.08) and high ACR (0.44) was the most adaptive for dry soils/drought (Fig. 3d). The significances of the root aerenchyma formation, along with stele and/or xylem development for tolerance to drought has been discussed previously (Uga *et al*., 2008; Zhu *et al*., 2010). Recently, small numbers of cortical cell files have also been proposed to be involved in efficient water uptake in dry soils (Chimungu *et al*., 2014). Our models include all these concepts comprehensively (Fig. 3d). As deep rooting systems are key for a tolerance of crops to drought (Uga *et al*., 2013), low CSR and high ACR could contribute to the development of deep rooting systems in wild Poaceae species. A wheat variety, however, having smaller xylem in the roots was previously found to be more tolerant to drought than that was another wheat variety having larger xylem (Richards & Passioura, 1989), suggesting that not only efficient water transport but also appropriate water use efficiency is essential for the growth in drought. A low XSR might be essential for the adjustment of water use efficiency in dryland species.

The adaptive root models for moderately high SWC (48%) and high SWC (81%) showed that a high CSR (7.98 and 13.04 for 48% and 81%, respectively) with high XSR (0.10 and 0.12, respectively) and with high ACR (0.39) should be adaptive to flooding (Fig. 3d). These results are consistent with previous findings showing that a large cortex (i.e. high CSR) with a large aerenchyma (i.e. high ACR) contributes to efficient internal oxygen diffusion from the shoot base to the root tips in flooding (Colmer, 2003; Yamauchi *et al*., 2019; Pedersen *et al*., 2021). Indeed, the greater potential to form aerenchyma in the roots of the wild relatives of maize is associated with an improved tolerance to soil flooding (Mano & Omori, 2013). Further, comparisons between the moderately high SWC and high SWC showed that a high CSR amplifies the AWR (0.35 and 0.37 for 48% and 81%, respectively) in the roots of a high‐SWC species, even though the ACR (0.39) was comparable to each other (Fig. 3d). This trend was more apparent with low SWC (16%) where the ACR (0.44) and AWR (0.31) were respectively higher and lower than those in high SWC (Fig. 3d).

In the present study, we investigated the species‐level differences of root anatomical traits of 18 wild Poaceae species in their natural habitats. From the results of the regression model analyses for the correlations of the root tissue ratio to the SWC, we predicted the optimal root anatomical traits that are essential for adaptations to the soil water gradients. As our concept includes the coordination of comprehensive root anatomical traits that contribute to plant adaptations from lowest to highest SWC, they will aid the improvement of many agronomically important crops for drought and flooding tolerances.

## Author contributions

TY conceived the idea, performed the experiments and analysed the data; TY and OP wrote the manuscript; TY designed the research with the contributions from OP, MN and NT.

## Supporting information


**Fig. S1** Methodology for the field survey of the root anatomical traits of the wild Poaceae species.
**Fig. S2** Root tissue areas of the wild Poaceae species.
**Fig. S3** Numbers and average areas of the xylem and aerenchyma in the roots of the wild Poaceae species.
**Fig. S4** Differences in plant height among the wild Poaceae species
**Fig. S5** Principal component analyses of the root anatomical traits of the wild Poaceae species.
**Fig. S6** Linear and nonlinear regression analyses of the soil water content and root tissue ratio of the wild Poaceae species.
**Fig. S7** Response of the root tissue ratio of the wild Poaceae species to the increased soil water content.
**Table S1** Soil water content in the surrounding of the wild Poaceae species after three nonrainy days or after three intermittent rainy days.
**Table S2** Principal component analyses of the root anatomical traits of the wild Poaceae species.
Please note: Wiley Blackwell are not responsible for the content or functionality of any Supporting Information supplied by the authors. Any queries (other than missing material) should be directed to the *New Phytologist* Central Office.
