# Supplementary material for: Key root traits of Poaceae for adaptation to soil water gradients
Source: New Phytol. 2020 Dec 20;229(6):3133–40. doi: 10.1111/nph.17093 (PMC7986152; doi:10.1111/nph.17093)
Supplement: Supplementary file 1 — Fig. S1 Methodology for the field survey of the root anatomical traits of the wild Poaceae species. Fig. S2 Root tissue areas of the wild Poaceae species. Fig. S3 Numbers and average areas of the xylem and aerenchyma in the roots of the wild Poaceae species. Fig. S4 Differences in plant height among the wild Poaceae species Fig. S5 Principal component analyses of the root anatomical traits of the wild Poaceae species. Fig. S6 Linear and nonlinear regression analyses of the soil water content and root tissue ratio of the wild Poaceae species. Fig. S7 Response of the root tissue ratio of the wild Poaceae species to the increased soil water content. Table S1 Soil water content in the surrounding of the wild Poaceae species after three nonrainy days or after three intermittent rainy days. Table S2 Principal component analyses of the root anatomical traits of the wild Poaceae species. Please note: Wiley Blackwell are not responsible for the content or functionality of any Supporting Information supplied by the authors. Any queries (other than missing material) should be directed to the New Phytologist Central Office. [file NPH-229-3133-s001.pdf]

New Phytologist

New Phytologist **Supporting Information**

Article title: Key root traits of Poaceae for adaptation to soil water gradients.
Authors: Takaki Yamauchi, Ole Pedersen, Mikio Nakazono and Nobuhiro Tsutsumi
Article acceptance date: 12 Nov 2020

The following Supporting Information is available for this article:

Fig. S1 Methodology for the field survey of the root anatomical traits of the wild Poaceae species.

Fig. S2 Root tissue areas of the wild Poaceae species.

Fig. S3 Numbers and average areas of the xylem and aerenchyma in the roots of the wild Poaceae species.

Fig. S4 Differences in plant height among the wild Poaceae species.

Fig. S5 Principal component analyses of the root anatomical traits of the wild Poaceae species.

Fig. S6 Linear and non-linear regression analyses of the soil water content and root tissue ratio of the wild Poaceae species.

Fig. S7 Response of the root tissue ratio of the wild Poaceae species to the increased soil water content.

Table S1 Soil water content in the surrounding of the wild Poaceae species after three non-rainy days or after three intermittent rainy days.

Table S2 Principal component analyses of the root anatomical traits of the wild Poaceae species.

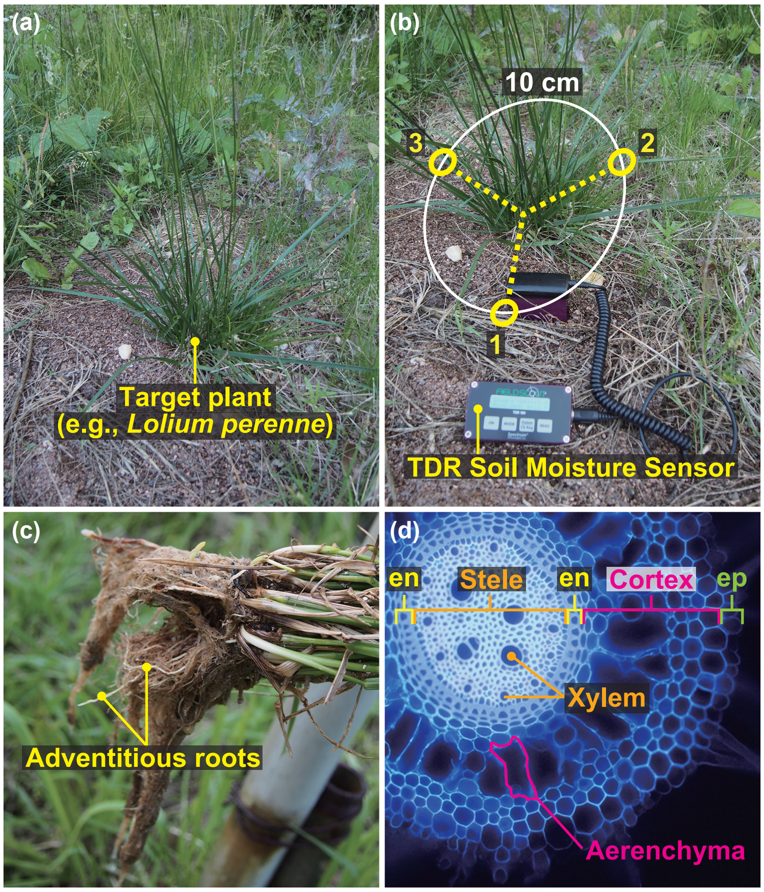

Fig. S1 Methodology for the field survey of the root anatomical traits of the wild Poaceae species. (a) Target species were identified based on their morphological features, such as panicles and stem trichomes. (b) The soil water content at a depth of 10 cm below the soil surface in the surroundings of the target plants was measured using a TDR soil moisture sensor. The measurements were conducted three times at 10 cm from the target plants. (c) The roots of the target plants were washed, and the whitish young adventitious roots (100 mm- to 150 mm-lengths) were sampled. (d) Root cross sections were prepared at 10 mm from the root-shoot junction of adventitious roots by hand sectioning with a razor blade. The boundary of stele and cortex, i.e. endodermis, was detected under UV irradiation. en; endodermis, ep; epidermis.

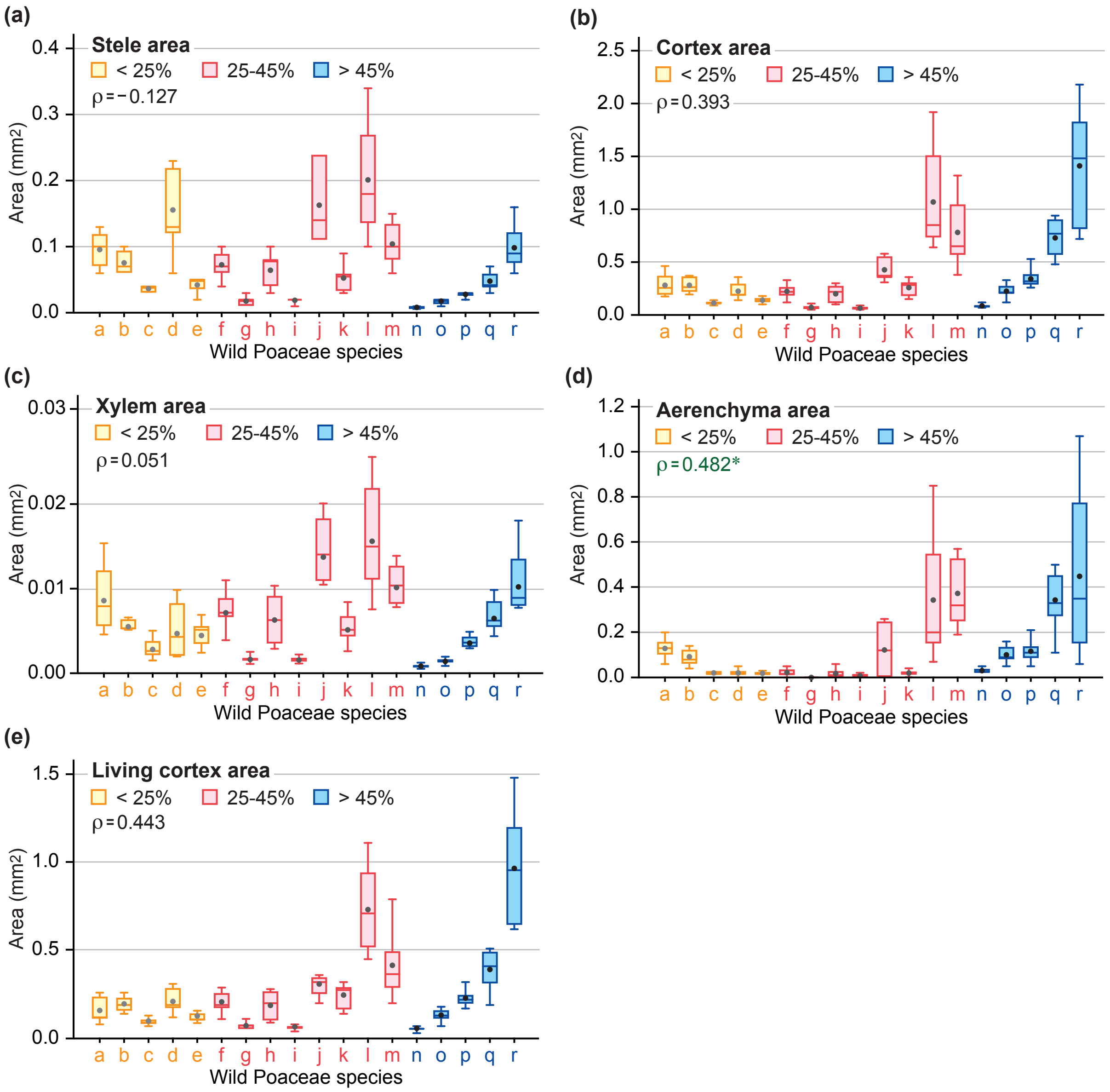

Fig. S2 Root tissue areas of the wild Poaceae species. The areas of the stele (a), cortex (b), xylem (c), aerenchyma (d) and living cortex (e) at 10 mm from the root-shoot junctions of the adventitious roots of the wild Poaceae species. The results of the Spearman' s rank correlation tests (ρ-value; *P* < 0.05 is indicated by *) between the soil water content and the area of each root tissue are shown in the upper left of each graph. Boxplots show the median (horizontal lines), 25th to 75th percentiles (extension of the boxes), minimum to maximum values (error bars) and mean values (dots in the boxes) (n = 6 to 9). The species represented by letters (a to r) are as defined in Table 1.

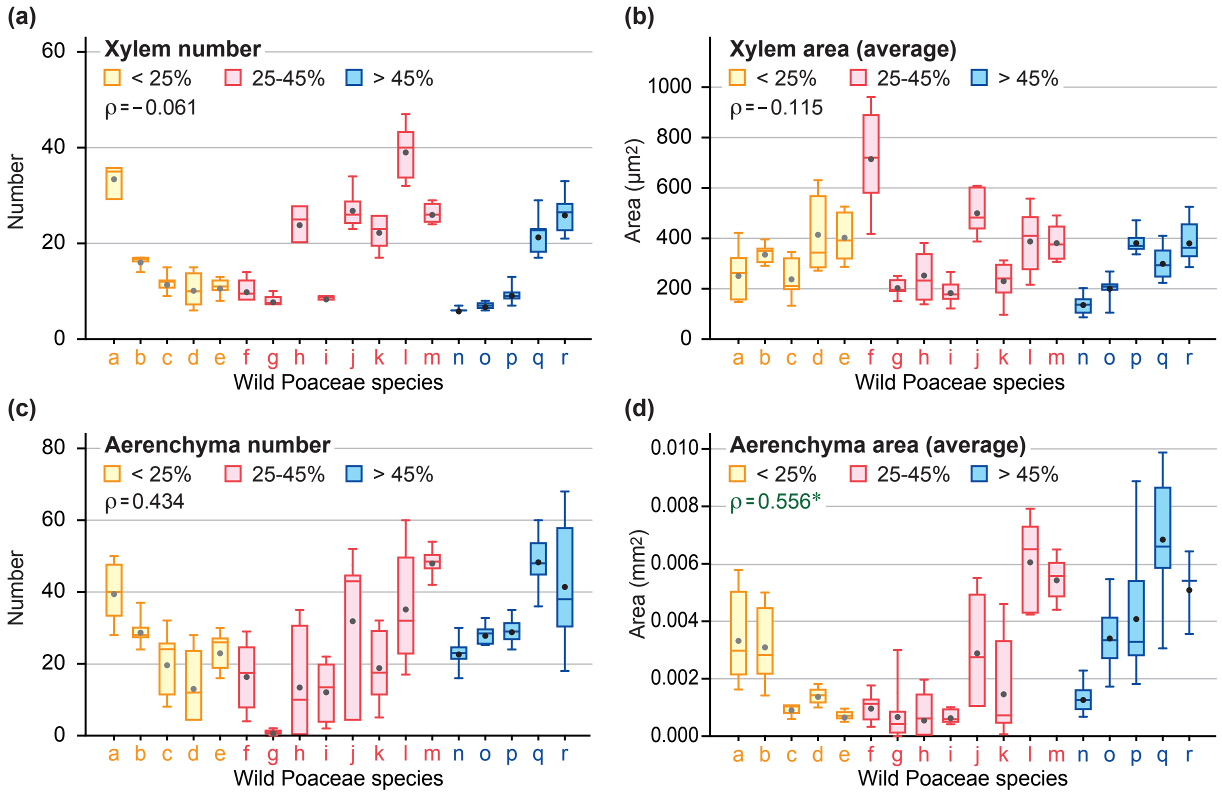

Fig. S3 Numbers and average areas of the xylem and aerenchyma in the roots of the wild Poaceae species. Number of Xylem (a), average area of xylem (b), number of aerenchyma (c) and average area of aerenchyma (d) at 10 mm from the root-shoot junction of the adventitious roots. The results of the Spearman' s rank correlation tests (ρ-value; $P < 0.05$ is indicated by *) between the soil water content and the number or area of the xylem or aerenchyma are shown in the upper left of each graph. Boxplots show the median (horizontal lines), 25th to 75th percentiles (extension of the boxes), minimum to maximum values (error bars) and mean values (dots in the boxes) (n = 6 to 9). The species represented by letters (a to r) are as defined in Table 1.

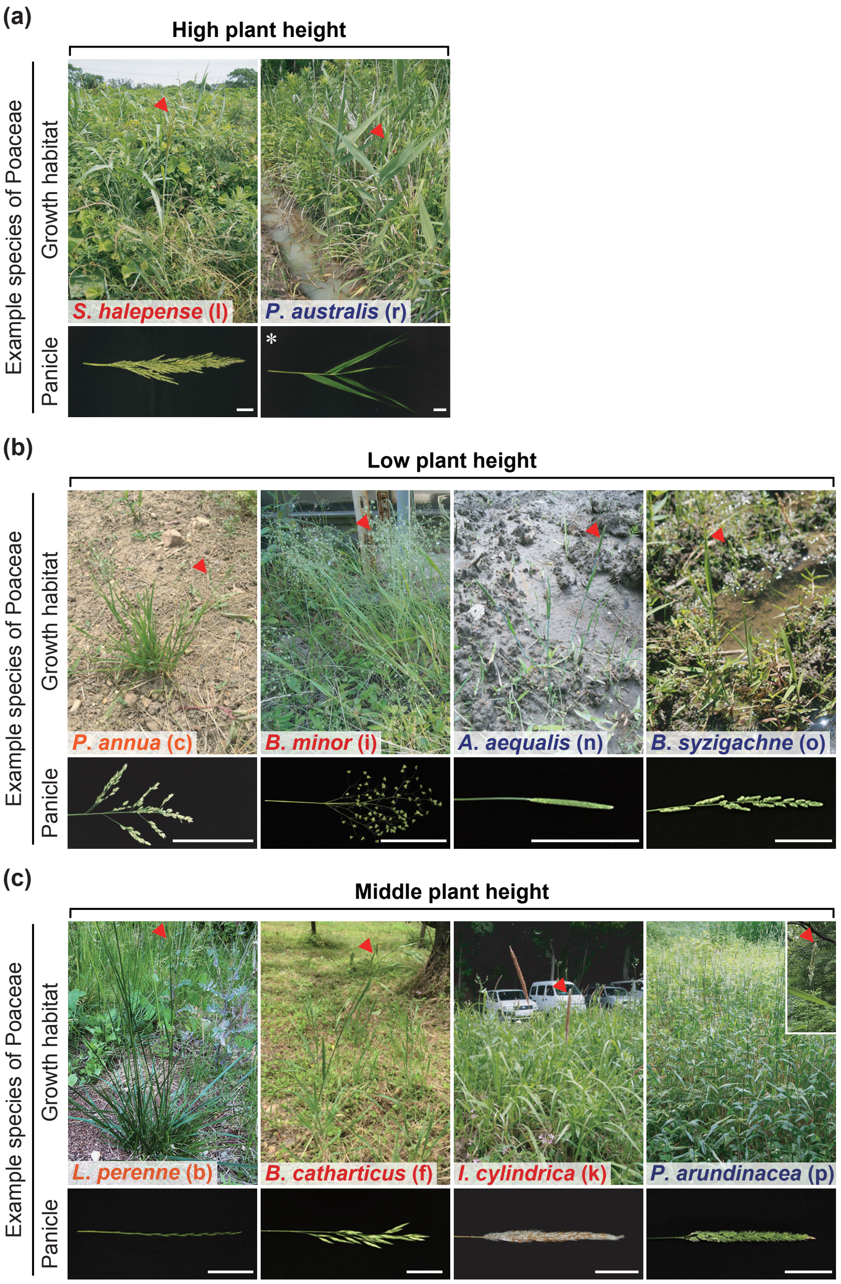

Fig. S4 Differences in plant height among the wild Poaceae species. Example photos of 10 of the 18 target species in their natural habitats and the panicles (or leaves) of sampled plants. Leaves were photographed for *Phragmites australis* as it was sampled before heading (shown as asterisk). Bars = 5 cm. Red arrows indicate the sampled panicles or leaves. The 10 species were classified into high (a), low (b) and middle (c) plant-height groups according to the panicle and leaf sizes.

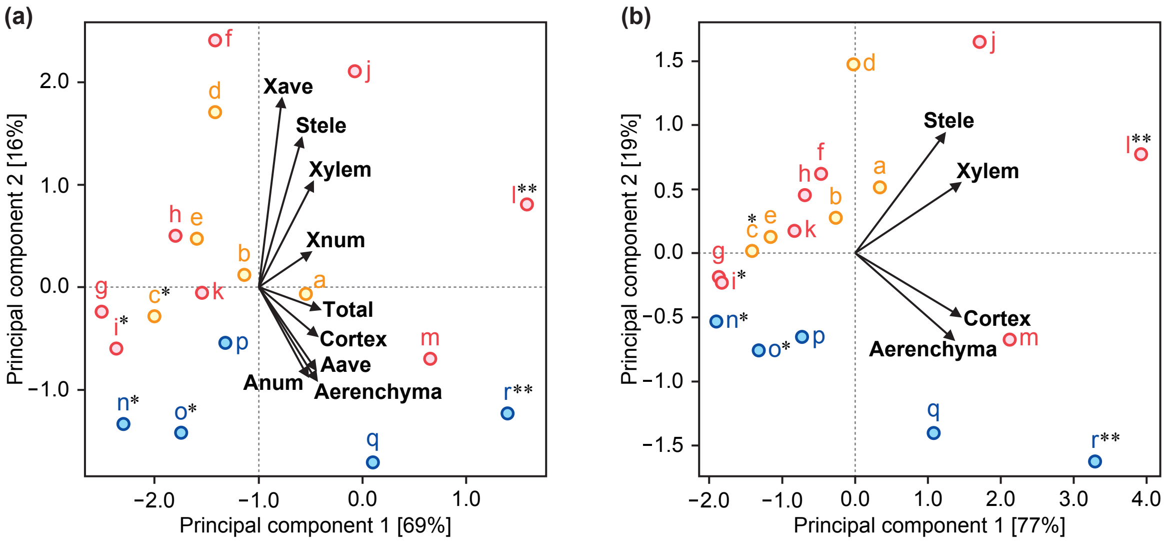

Fig. S5 Principal component analyses of the root anatomical traits of the wild Poaceae species. (a) The map defined by principal components 1 and 2 (69% and 16% of the variances, respectively) of the principal component analysis (PCA) performed on the total cross-sectional area (Total), areas of stele, cortex, xylem and aerenchyma, numbers of xylem (Xnum) and aerenchyma (Anum) and average areas of the xylem (Xave) and aerenchyma (Aave). (b) The map defined by principal components 1 and 2 (77% and 19% of the variances, respectively) of the PCA performed on the areas of the stele, cortex, xylem and aerenchyma. Arrows indicate loadings of the variables to the first and second principal components. The species having lower and higher plant heights are shown as * and **, respectively (shown in Fig. S4). The other species have middle plant heights. The species represented by letters (a to r) are as defined in Table 1.

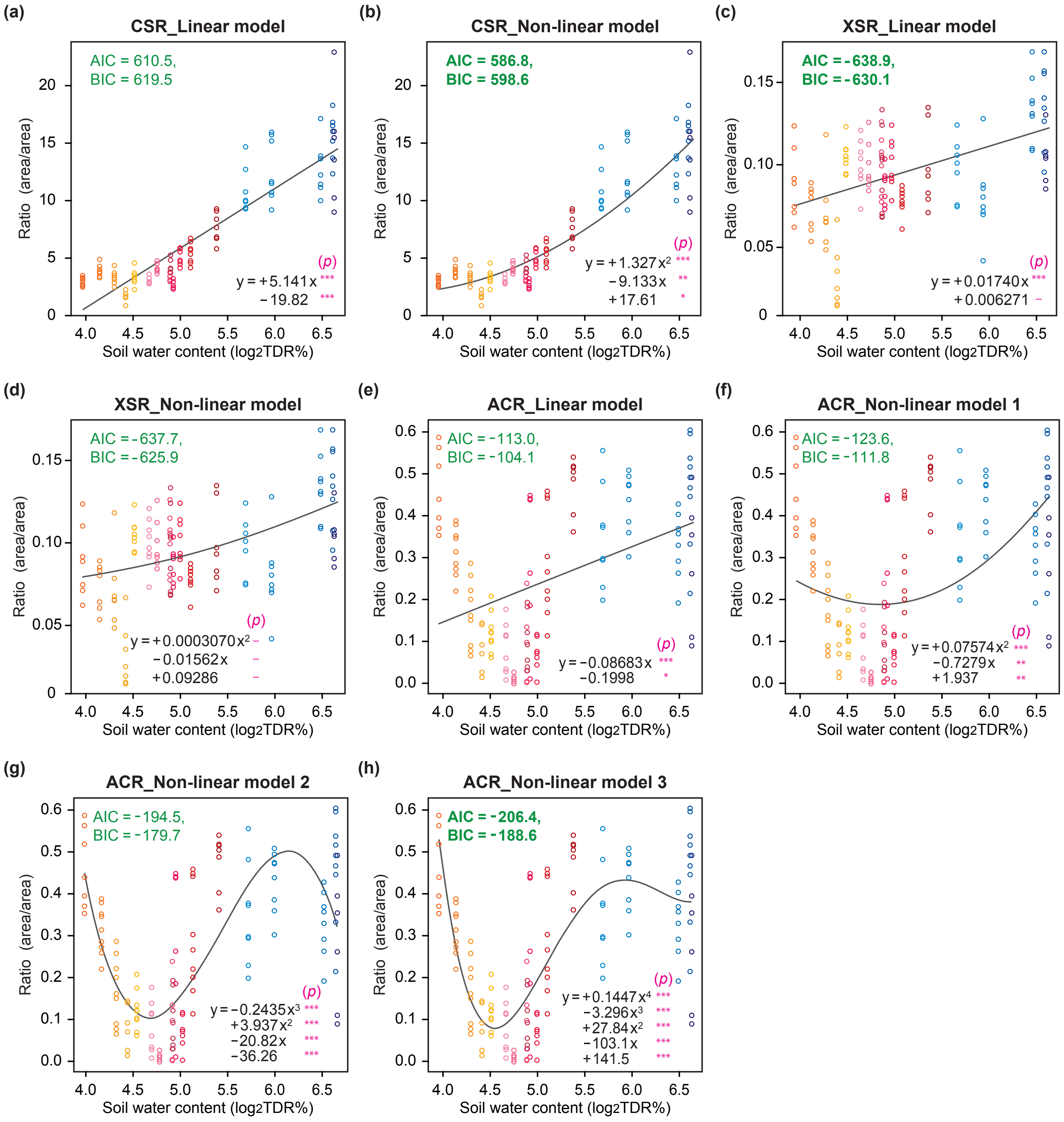

Fig. S6 Linear and non-linear regression analyses of the soil water content and root tissue ratio of the wild Poaceae species. Linear regression analyses of the soil water content (SWC; log2TDR%) and cortex to stele ratio (CSR) (a), xylem to stele ratio (XSR) (c) or aerenchyma to cortex ratio (ACR) (e). Nonlinear regression analyses of SWC and CSR (b), XSR (d) or ACR (f-h). The solid gray line shows the models obtained by the regression analyses. The coloured dots indicate the values of CSR, XSR and ACR in the adventitious roots of each wild Poaceae species. The Akaike information criterion (AIC) and Bayesian information criterion (BIC) model fit statics were used for the model selections. The statistical significances of each term in equations at $P < 0.001$, $P < 0.01$ and $P < 0.1$ are denoted by ***, ** and *, respectively.

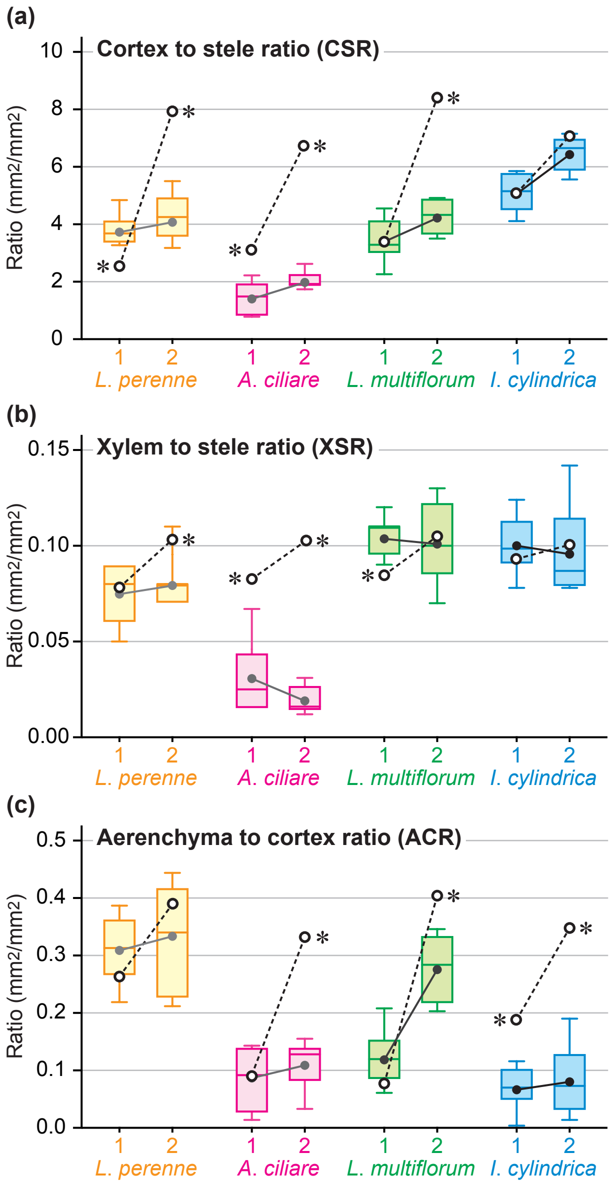

Fig. S7 Response of the root tissue ratio of the wild Poaceae species to the increased soil water content. Roots of the target plants were sampled after three continuous non-rainy days (shown as no. 1) or after three intermittent rainy days (shown as no. 2). Cortex to stele ratio (CSR) (a), xylem to stele ratio (XSR) (b) and aerenchyma to cortex ratio (ACR) (c) at 10 mm from the root-shoot junction of the adventitious roots of the four wild Poaceae species. Boxplots show the median (horizontal lines), 25th to 75th percentiles (extension of the boxes), minimum to maximum values (error bars) and mean values (dots in the boxes) (n = 6 to 9). Solid lines connecting filled circles (i.e. mean values) indicate the actual responses of root tissue ratio to the intermittent rainfall. Dashed lines connecting unfilled circles indicate the differences in the root tissue ratio calculated by the regression models (Fig. 3), which were constructed based on the species-level differences of the 18 wild Poaceae species, using the soil water content before and after rainfall (Table S1) as explanatory variables. The statistical significances between the actual values and calculated values at $P < 0.001$ are denoted by * (one-sample *t*-test).

Table S1 Soil water content in the surrounding of the wild Poaceae species after three non-rainy days or after three intermittent rainy days

Whether	Subfamily	Species	Soil water content (% TDR)[a]	Adventitious root length (mm)[b]
non-rainy	Pooideae	*Lolium perenne* #1	17.6 ± 1.8	71.9 ± 14.8
rainy	Pooideae	*Lolium perenne* #2	47.7 ± 2.5	65.9 ± 16.7
non-rainy	Pooideae	*Agropyron ciliare* #1	21.3 ± 1.5	66.7 ± 11.1
rainy	Pooideae	*Agropyron ciliare* #2	41.3 ± 1.6	61.8 ± 19.4
non-rainy	Pooideae	*Lolium multiflorum #1*	22.8 ± 8.8	78.8 ± 11.7
rainy	Pooideae	*Lolium multiflorum* #2	51.1 ± 2.8	62.4 ± 17.3
non-rainy	Panicoideae	*Imperata cylindrica* #1	31.9 ± 1.6	67.0 ± 15.8
rainy	Panicoideae	*Imperata cylindrica* #2	42.8 ± 3.4	65.0 ± 14.1

[a]Values are means ± SE (n = 3). [b]Values are means ± SE (n = 6-9).

Table S2 Principal component analyses of the root anatomical traits of the wild Poaceae species

	**Experiment 1**		**Experiment 2**	
	PC1	PC2	PC1	PC2
Standard deviation	2.50	1.18	1.76	0.87
Proportion of variance	69%	16%	77%	19%
Whole root area	0.39	-0.07	-	-
Stele area	0.29	0.48	0.46	0.65
Cortex area	0.38	-0.16	0.53	-0.40
Xylem area	0.35	0.34	0.52	0.35
Aerenchyma area	0.37	-0.29	0.50	-0.54
Xylem number (Xnum)	0.33	0.11	-	-
Aerenchyma number (Anum)	0.32	-0.29	-	-
Average xylem area (Xave)	0.15	0.61	-	-
Average aerenchyma number (Aave)	0.37	-0.27	-	-
